# Delivering care under uncertainty: Swiss providers’ experiences in caring for women with spinal cord injury during pregnancy and childbirth – an expert interview study

**DOI:** 10.1186/s12884-016-0976-y

**Published:** 2016-07-22

**Authors:** Sue Bertschy, Jürgen Pannek, Thorsten Meyer

**Affiliations:** Swiss Paraplegic Research (SPF), Guido A. Zäch-Strasse 4, CH-6207 Nottwil, Switzerland; Department of Health Sciences and Health Policy, University of Lucerne and SPF, Nottwil, Switzerland; Swiss Paraplegic Centre (SPZ), Neuro-Urology, Guido A. Zäch-Strasse 2, CH-6207 Nottwil, Switzerland; Hannover Medical School, Institute for Epidemiology, Social Medicine and Health Systems Research, Carl-Neuberg-Str. 1, D-30625 Hannover, Germany

**Keywords:** Pregnancy, Childbirth, Spinal cord injury, Care provider, Health professionals, Interdisciplinary collaboration, Woman, Rare event

## Abstract

**Background:**

When different health problems such as pregnancy and spinal cord injury (SCI) occur together, providing adequate care becomes even more challenging. Women with SCI may encounter a variety of specific problems and symptoms during pregnancy and childbirth, including urinary tract infections, pressure ulcers, constipation, autonomic dysreflexia, and preterm labour. Therefore, expertise from different medical specialties, especially spinal cord medicine and gynaecology are required. What is totally normal for experts of one specialty could cause a problem for experts from another specialty. Therefore, this study aimed to reconstruct the perceptions and experiences of healthcare providers in Switzerland in caring for women with SCI during pregnancy and childbirth.

**Methods:**

The perception and experience of healthcare professionals toward providing care for women with SCI during pregnancy and labour were elicited using qualitative expert interviews and analysed using grounded theory techniques. Fifteen health professionals were interviewed, including gynaecologists (*n* = 4), midwives (*n* = 3), physical medicine and rehabilitation professionals (*n* = 4), urologists (*n* = 3), and a peer counselor (*n* = 1).

**Results:**

Care delivery experiences of health professionals could be described as a forced reaction to decision making under uncertainty. However, health professionals seemed to express three different attitudes while handling the situation: (i) protective concerned attitude, (ii) ‘no big deal’ attitude, or (iii) precautionary attitude. The applied strategies were influenced by the conditions of the healthcare system, policies in place, and health professionals’ behaviours. Consequently, health professionals faced with uncertainty felt like actors in a fragmented treatment process and called for interdisciplinary collaborations.

**Conclusions:**

Our findings highlight the diversity of perspectives among different healthcare professionals with respect to the approach to care and delivery services for pregnant women with SCI. A need for more specific services, information, guidance, and guidelines for health professionals caring for woman with SCI during pregnancy and childbirth was identified. We strongly recommend further research on the development of integrated care concepts as well as clinical studies for establishing a more profound knowledge base.

**Electronic supplementary material:**

The online version of this article (doi:10.1186/s12884-016-0976-y) contains supplementary material, which is available to authorized users.

## Background

An increasing number of women with SCI decide to become mothers [1–3]. Approximately 14 % of women with SCI in the US have children after their injury [4, 5]. Thus far, there are no records of the number of women who gave birth after SCI in Switzerland. Until today, becoming a mother with SCI is rare and knowledge in this field is relatively minimal [6, 7]. Therefore, healthcare for pregnant women with SCI is unique and requires special medical attention.

Pregnancy and childbirth in women with SCI can cause specific problems and symptoms, which can vary from woman to woman. For instance, clinical studies have documented an elevated risk of developing urinary tract infections, pressure ulcers, constipation, autonomic dysreflexia, and thrombosis [1, 8–14]. In addition, various obstetrical challenges, including preterm labour, unattended delivery, and premature births, may arise [15–18]. Women with SCI usually take medications for chronic conditions such as spasms and bladder and bowel problems, or for pain management [1, 16, 19]. When different healthcare needs arise together such as in SCI patients who are pregnant, providing adequate care becomes even more demanding.

Studies with health professionals providing care for women with SCI during pregnancy and childbirth indicate a scarcity of relevant studies [6, 10, 20], missing recommendations based on clinical trials [5], little availability and accessibility of reproductive services [7, 14, 21, 22], and substantial deficits in interprofessional collaboration [11, 14, 23] to achieve successful pregnancies with healthy outcomes for the mother and child.

Providing care for those women requires a combined knowledge in gynaecology and SCI rehabilitation. It seems that these distinct skills very rarely run together [14, 24]. Therefore, health professionals from different fields are usually involved in the care process. This requires a special approach toward collaboration and integration of care [5]. What is a completely normal approach for one specialisation, could cause a problem for the other specialisation, e.g., administering antibiotics, performing obstetric ultrasonography, or prescribing thrombosis prophylactics may have unintentional harmful effects in pregnant women with SCI. Thus, health care services need to integrate multiple professional perspectives [1, 25, 26].

Reproductive health services for able-bodied pregnant women in Switzerland are sufficiently accessible and available [27]. However, in a previous study we observed that women with SCI face various difficulties in accessing care and in finding care providers with knowledge of both specialities—spinal cord medicine and gynaecology. It seems that so far there is no specialised pregnancy counselling service. The lack of specific comprehensive knowledge, cooperation, and regulations may cause confusion, as specific guidelines in Switzerland have not been implemented in one field [14]. As described above, this could cause a source of tension for some reproductive and SCI health care providers.

The question of how professionals deal with these challenges remains. In this study, we aimed to reconstruct the perceptions and experiences of healthcare providers in caring for women with SCI during pregnancy and childbirth. Our goal was to identify possible deficiencies in healthcare services, pinpoint probable reasons for it, and suggest ways to overcome these challenges.

## Methods

### Study design

Between May and September 2014, we conducted a study with health professionals from the healthcare communities and hospital settings in Switzerland. Semi-structured individual expert interviews [28] were conducted with health professionals from various specialisations who provided care for woman with SCI during pregnancy and childbirth. The providers were recruited through a previous study in which mothers with SCI had provided the names of their treating health care provider during pregnancy [14] and snowballing in the medical community. For reasons of analysis, only German-speaking health care professionals were included.

While we could have restricted our analysis to summarize what the experts reported during interviews, during the course of our investigation, it became clear that it was worthwhile to develop a deeper understanding of these special healthcare situations. Therefore, we decided to reconstruct the experiences and perceptions of the healthcare providers and attempted to develop possible reasons for and consequences of their experiences. To achieve this, we resorted to analysis techniques developed within the framework of grounded theory (GT) methodology [29] for gaining a deeper understanding of these challenges. Considering the sampling strategy, the GT methodology was only applied in the analysis section.

### Study participants and recruitment

**Fig. 1:**
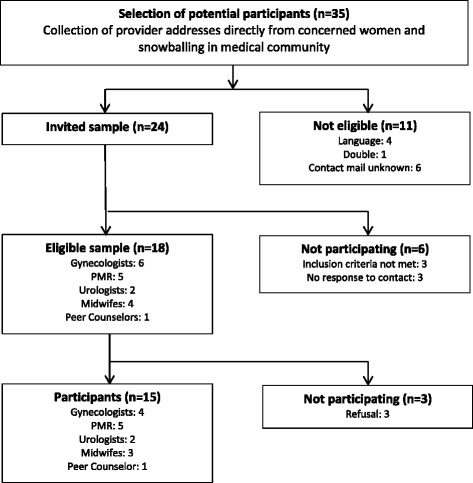
Recruitment of participants

The study included German-speaking healthcare providers in Switzerland who treated at least one pregnant woman with SCI during her pregnancy or childbirth. In Switzerland, there is no registry or official list of care providers who offer such services. Therefore, we used information from a previous study with concerned mothers [14]. We identified six main professional groups who accompanied women with SCI during pregnancy and childbirth, and a selective sample [30] was obtained from these groups for data collection: gynaecologists, physical medicine and rehabilitation (PMRs) professionals, urologists, midwifes, anaesthesiologists, and peer counsellors. We aimed to conduct 15 interviews, with no more than 5 interviews per healthcare provider group. A total of 35 potential participants were identified and 24 were contacted by email, phone, or in person. Due to reasons of language, double address, and no identification possible (unknown contact address), 11 health care providers were excluded. Of the 24 healthcare providers, 3 did not meet the inclusion criteria and 3 did not respond to our emails and phone calls. Of the remaining eligible sample of 18 healthcare providers, 3 refused to participate because of time constraints. Subsequently, 15 interviews were conducted with the following distribution of participants: 5 PMR professionals, 4 gynaecologists, 3 midwives, 2 urologists, and 1 peer counselor (Fig. 1).

We faced difficulties finding anaesthesiologists during data collection: either the mothers did not remember their anaesthesiologist’s name or we were unable to find the anaesthesiologist’s location. In every interview, we asked interviewees for further possible participants who matched the inclusion criteria. We also asked health professionals if they knew any anaesthesiologists involved in the care of SCI women during pregnancy and childbirth. Either they did not remember who was responsible for anaesthesia or the anaesthesiologist had left the hospital. Therefore, we excluded this group from the study. In Switzerland, the medical management of persons with SCI is mainly provided by PMR specialists; therefore, we did not include neurologists in our study.

Interviews were conducted in the form of semi-structured interviews, guided by previously developed key questions concerning their experiences with pregnant women with SCI, the main challenges they faced, how they gained their expertise in pregnancy with SCI, and how collaboration with other colleagues was performed or perceived. The full interview guide can be seen in the Additional file 1.

All interviews were conducted by either the first author or a research assistant trained in qualitative research methods. Since the experts’ jobs and busy schedules often led to time constraints if interviews were planned, we offered them the possibility of a phone interview if this was more suitable to their schedule. Nine participants opted for a phone interview, while the other six were interviewed at their workplace. The interview durations ranged from 16 to 51 min, with an average of 28 min.

Prior to the interview session, participants received study information and an invitation letter by email or post. At the interview session itself, the interviewer repeated information about study aim, procedure, and how the authors protected the anonymity and confidentiality of the participants. The participants had the right to withdraw from our study at any time. All participants’ names were replaced with a pseudonym. Interviews were audio-taped, transcribed, and analysed verbatim in German. Responses that represented variation within each theme were selected from the transcripts for inclusion in this article and translated from German into English.

The study was conducted in accordance with the ethical principles of the Declaration of Helsinki [31]. The participants were informed about the aims of the research project and took part in the study voluntarily as part of their professional commitment. Oral informed consent was obtained and recorded before starting the interviews (Tables 1 and 2).

**Table 1:** Study participants

Groups	Setting
Group 1	
Gynaegologist	Primary Care Facility (2)
	Private Practice (1)
	District Hospital (1)
Group 2	
Midwifes	Independent (2)
	Private Practice (1)
Group 3	
PMR professionals	Outpatient Clinic (1)
	SCI Rehab Clinic (2)
	University Hospital (1)
	Paraplegic Association (1)
Group 4	
Urologists	Outpatient Clinic (2)
Group 5	
Peer counselor	SCI Rehab Clinic (1)

**Table 2:** Participants characteristics

	Pseudonym	Function	Field of expertise	Setting	Experience with pregnant women with SCI
1	Matthias Gerber	Senior physician	Gynaecology	District Hospital	Treatment of patients during pregnancy who have been sent to the hospital from the surrounding area and SCI centre; gained knowledge through interdisciplinary exchange of knowledge with colleagues and high interest in SCI; practice located close to an SCI centre; participates in workshops about sexuality with SCI.
2	Michael Brunner	Chief resident	Urology	SCI Outpatient Clinic	Treatment of patients during pregnancy for the past 7 years; high competence in treating urological problems in SCI patients.
3	Anton Denner	Online-Doctor	Spinal Cord Medicine/Internist	Paraplegic Association	Online consultations for SCI-related questions; no gynaecological knowledge.
4	Nicole Müller	Senior physician	Spinal Cord Medicine/ Internist	SCI Rehab Clinic	Treatment of 1 patient during pregnancy; longstanding experience in treating SCI patients; patient suffered from SCI right after getting pregnant; discussions about treatment with her team and the treating gynaecologist.
5	Martin Roggo	Co-Chief resident	Gynaecology	Primary care facility	Treatment of 2 disabled patients during pregnancy; one doesn’t depend on a wheelchair anymore.
6	Franziska Schneider	Midwife	Midwife	Private Practice	Treatment of 1 patient during pregnancy and birth; work experience: 5 years.
7	Julia Peter	Chief resident	Spinal Cord Medicine	University Hospital	Treated and accompanied 10 patients during pregnancy; longstanding experience in treating SCI patients; comparatively less expertise in gynaecology.
8	Barbara Jung	Senior physician	Spinal Cord Medicine	SCI Rehab Clinic	Treatment of several patients during pregnancy; last treatment of pregnant women with SCI was a couple of years ago.
9	Anna Weiss	Midwife	Midwife	Independent/Primary care facility	Treatment of 1 patient during pregnancy; work experience: 13 years; wrote a thesis about pregnant women with SCI by interviewing 2 women with SCI.
10	Jasmin Rieger	Peer Counsellor	Peer counselor	SCI Outpatient Clinic	Accompanied patients during pregnancy as a peer, experienced pregnancy and SCI herself; works in an SCI environment; high interdisciplinary exchange with colleagues and other peers; conducts workshops on sexuality and SCI on a regular basis.
11	Katharina Bach	Midwife	Midwife	Independent	Treatment of 1 patient during pregnancy; patient was a friend and she accompanied her mainly during puerperal visits and had some contact during pregnancy; work experience: 5 years; knows the SCI community well.
12	Elisabeth Frey	Chief resident	Gynaecology	Primary care facility	Treatment of 5–10 patients during pregnancy; work experience: 30 years in larger clinics in Switzerland and other countries; gained knowledge about SCI through interdisciplinary exchange with colleagues and literature.
13	Georg Fischer	Head of SCI Outpatient Clinic	Spinal Cord Medicine	SCI Outpatient Clinic	Accompanied patients during pregnancy; no knowledge in gynaecology; treated SCI-related problems and consulted patients and gynaecologists.
14	Alexander Brandt	Senior physician	Urology	SCI Outpatient Clinic	Treatment of 3 patients during pregnancy; urologist since 6 years; worked in Switzerland and other countries in SCI clinics; treated SCI-related problems and diagnosed one pregnancy during examination for SCI-related problems.
15	Jonathan Steiner	Senior physician	Gynaecology	Private Practice/External physician with hospital affiliation	Treatment of 1 patient during pregnancy; knowledge gained mainly through literature and online research.

### Data analyses

We resorted to analytical techniques developed within the framework of GT, acknowledging that different traditions of GT exist [32]. Our analysis was informed by the constructivist approach of Strauss and Corbin [33]. The Straussian approach to data analysis suggests a process of comparing concepts and their relationships. Moreover to pay attention to wider contextual factors that can influence a situation. Using GT analysis to data allows inductive category development as opposed to impose predefined categories on the data.

Accordingly, the interview transcripts were analysed using open and axial coding [29]. We focussed on scrutinising interview transcripts line by line to explore concepts that appeared in the data and writing first memos about conceptual and theoretical ideas that emerged during the course of the analysis. We repeatedly went through the interviews and merged open codes and memos into possible categories. The combination of open coding with increasingly focussed procedures contributed to the development of a deeper understanding of the content and meaning of the text [33]. The open coding was done independently by the first author (SB) and the research assistant. After open coding, coder checking occurred by engaging in an iterative process of refinement of categories between the two initial coders and co-authors (JP and TM).

During the process of axial coding, which was our second step, we used the ‘paradigm model’ of Strauss and Corbin [29]. We coded for similarities and differences in the data, which involved constantly comparing indicators and concepts, which in turn led to new concepts [33]. The goal during this process was to elaborate the central categories for the theory on the experiences of the health professionals in caring for pregnant women with SCI and their different expressions in the data. The main aim of this step was to identify the *central phenomenon* of the data. Our study participants had certain ways in which they dealt with the phenomenon, which we listed under the term *strategies*. The strategies then had certain *consequences*. The listed *contextual conditions* are coded (according to Strauss and Corbin’s suggested practise) as belonging to the central phenomenon. Each contextual condition should represent the particular set of conditions that provide a framework in which the phenomenon under study takes place. Within the structural conditions are *actions*, which are referred to as the *intervening conditions* [33]. The axial coding was performed by the first author in regular meetings with the last author using a critical perspective with focus to the action and interaction strategies of the experts.

The study’s *internal validity* [34] was enhanced by triangulation of different professional perspectives from the research team that consisted of a social researcher (first author, SB) and paraplegic women with a special emphasis on women’s views [1, 14, 20]; a research assistant with background in health sciences and policy, the second author (JP) with leading experience in the field of urology working in a SCI rehab clinic [1, 35]; and a senior researcher (last author, TM) experienced in qualitative and health services research [36, 37]. This procedure helped ensure that conclusions drawn from the authors were not based on a single perspective and helped to specify the resulting categories by an iterative reflection of the empirical data. Due to the small sample size and size variation in the groups, the results are mostly discussed as a whole, without addressing specific differences, similarities, or commonalities in between the groups.

We used MAXQDA10 for the open coding process and the visual software programme Mindjet 14 for developing and visualizing the categories and subcategories. Mindjet 14 allowed us to manage large amounts of data in a direct and uncomplicated way.

## Results

**Fig. 2:**
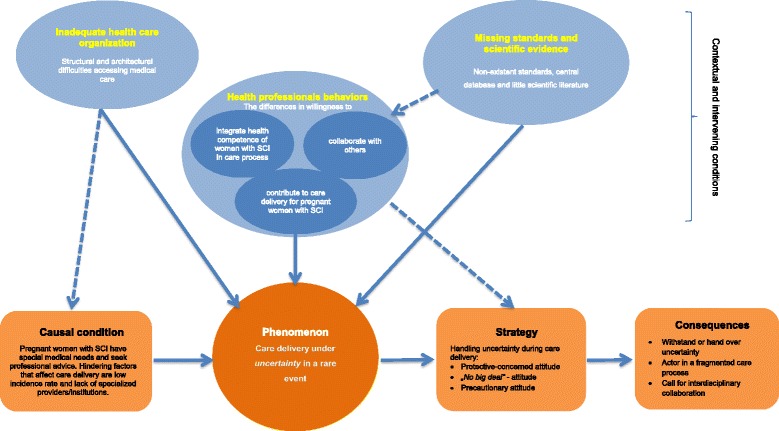
Delivering care under uncertainty in pregnant women with SCI (Coding paradigm adapted from Strauss and Corbin, 1996)

Coding data obtained from the 15 interviews provided a number of categories that could be arranged under a core topic (Fig. 2). The core category was derived from the service providers’ experiences while caring for women with SCI during their pregnancies and births. The way the service providers treated their patients was largely influenced by how they conceived their roles and their feelings of responsibility.

The treatment of women during this rare event presented a special challenge for the service providers. Our interpretation is that the service providers felt uncertain when treating these women. This uncertainty endangers an individual’s ability to treat and function optimally, which is closely associated with the ability to control and predict situations. Service providers’ experiences provide information about how efforts to regain certainty were initiated. The following elucidates the area of conflict among the treatment scenarios by means of different interconnected aspects resulting from the individual categories.

### Causal conditions

Every health professional mentioned the absence of a contact point for pregnant women with SCI. They were not able to name any individuals or institutions that offer explicit services or consultations for pregnant women with SCI. We assume that the rarity of the event is, due to its small incidence, also characterized by the absence of specialised providers.

*‘I think it is [the event] simply too rare. There is no one who really knows how it works best.’ Jonathan Steiner, gynaecologist*

The health professionals also highlighted further reasons that could lead to the phenomenon, such as progress in the field of medicine, increasing number of specialisations, and high expectations from pregnant paraplegic women vis-á-vis the healthcare system, resulting in these women having increased interactions with different healthcare professionals.

*‘…that (pregnant women with SCI) is (a) relatively rare (phenomenon), and I believe that knowledge isn’t so abundant among urologists and gynaecologists that one can say that everybody can treat it.’ Alexander Brand, urologist*

### Phenomenon

We identified *care delivery under uncertainty in a rare event* as the core category. The structural supply gaps described under the causal condition force service providers to provide treatment that is unfamiliar due to the complexity of care for pregnant women with SCI. The service provider must take many more factors into consideration than when treating an able-bodied woman. The attending physicians had neither the experience nor the competency to be able to comprehend all these factors and predict possible interactions, which creates a feeling of uncertainty. Everyone wanted to be able to precisely appraise what will happen during treatment. The interviewees expressed this by stating their uncertainty. However, not every participant spoke about his/her uncertainty or described this conflict. Based on the described patterns, we interpreted the service providers’ behaviour as coping mechanisms in the face of uncertainty. This uncertainty was observed among individuals and can be attributed to others. We assume that this uncertainty characterizes the experience of all the professionals interviewed. The role of a decision maker is often assigned to service providers, thus they have no choice but to accept the challenge facing them. Decisions had to be made, however, not only on the basis of current knowledge but also on missing knowledge and uncertainty. Unfortunately, many service providers face missing knowledge and uncertainty when dealing with women with SCI, which created an undesired and unpleasant situation for both the pregnant women and their providers.

*‘One notices (it) a bit among midwives. I also noticed it among physicians, that enormous fear or maybe ignorance exists about caring for the women. I wish that it would be actually accepted as an open topic. That the women (in wheelchairs) would be greeted just as openly as women who are not in a wheelchair. Because they deserve exactly the same care as the others. One need not be afraid.’ Anna Weiss, midwife*

In the following example, we describe a situation to illustrate the perceived uncertainty. A urologist explains to a pregnant woman with SCI how her gynaecologist could conduct a prenatal exam. We attribute the gynaecologist’s refusal to take over treatment to his/her fear of causing possible harm to the woman or child due to insufficient knowledge.

*‘I can remember one (pregnant woman with SCI) quite well who asked me how a prenatal exam is carried out because the gynaecologists did not dare to conduct the gynaecological examination. She was tetraplegic, and they were afraid that they would induce autonomic dysregulation if they performed a vaginal examination.’ Michael Brunner, urologist*

### Contextual and intervening conditions

#### Health professionals

The willingness of service providers to treat pregnant women with SCI can be triggered, intensified, or weakened by different conditions and can influence service providers’ coping with uncertainty during treatment.

#### Differences in willingness to contribute to care delivery for pregnant women with SCI

From our point of view, as soon as one takes responsibility for other individuals and their concerns, the fear of mistakes or one’s own failure generally arise. Every health professional is exposed to this risk and must learn to deal with it. A health professional’s fear serves as an indicator of the degree to which s/he is consciously aware of the risks and how s/he deals with them professionally. This is related to not only the fear of a treatment mistake, but also fear of personal failure, which accompanies every medical action from the very beginning and leads to different degrees of willingness to deliver treatment, as our study participants have experienced in themselves or their colleagues.

*‘…among gynaecologists, reservations apparently exist against treating such cases. An affected woman told me that she had difficulty finding a gynaecologist who said that he felt competent to treat someone sitting in a wheelchair and being pregnant.’ Michael Brunner, urologist*

Differences in professional role perception and the accompanying willingness to take responsibility for treatments were observed among all participants. The interviewed gynaecologists saw their responsibility in the specialised and methodically appropriate treatment of gynaecological problems and their gynaecological services. Similar attitudes closely related to the tasks within their specialisation could also be identified among the interviewed midwives. They usually refer these women to specialists for paraplegia for their SCI problems. Specialists for paraplegia behaved differently. They were more concerned about the holistic well-being of the pregnant women and were aware of the urgency of their needs. We assumed that these differences in the service spectrums are anchored in the characteristics of the participants’ specialisations. Specialists like gynaecologists and midwives might consider themselves responsible for organ systems but not holistic para-gynaecological problem constellations. In contrast, those specialised in spinal cord medicine are trained in and confronted with interdisciplinary thinking.

*‘PMR professionals don’t only treat urologic problems—I have also treated acute cases of paraplegia. I have seen patients with decubitus; I have also treated problems in internal medicine among paraplegics. I think that medical science is a broad field and urologists are urologists and gynaecologists are gynaecologists. Period.’ Julia Peter, PMR professional*

Another factor that influences taking over responsibility is familiarity with the topic. We witnessed a difference between participants who had never come across spinal cord medicine in their normal daily routine and those who had an experience of one kind or another with spinal cord medicine. There were individuals that clearly declined responsibility for spinal cord treatment, and others with long-term experience in caring for pregnant women with SCI who had amassed a lot of experience in such cases.

*‘…the PMR professionals told me where they saw the problem from their point of view, and I tried to translate that for myself. When the PMR professionals said, “spasticity or vegetative dystonia”, I considered what that could mean for the pregnancy. I tried to observe the foetal development and the woman with SCI separately. I quickly noticed that the SCI actually had relatively little importance in the pregnancy and foetal development.’ Matthias Gerber, gynaecologist*

Occasionally, the suspicion was expressed that colleagues took over the complicated care of pregnant women with SCI for reasons of prestige.

*‘It is usually very interesting for the gynaecologists to care for such a woman, and they want to keep the woman. Self-interest also plays a role.’ Anna Weiss, midwife*

However, participants also expressed assumptions that colleagues would not trust themselves to take over such care. There were even signs of refusal to accept these complex tasks.

*‘I believe they are afraid of complications. Of complications with autonomic hyperreflexia or, for example, that complications would arise with the paraplegia, that there would somehow be a rupture. I think it is easy: if someone has no knowledge about it, one also is afraid. I can, of course, understand that.’ Anna Weiss, midwife*

*‘Anaesthesiologists know what to do during a caesarean section. In contrast, SCI with lumbar anaesthesia plus no sensitivity, I think that is just too precarious for them.’ Jasmin Rieger, midwife*

#### Differences in the willingness to cooperate

Inexperience in treatment modalities or possible undesired treatment results appear everywhere in healthcare, more so when the service provider is confronted with situations in which crucial decisions must be made. The important aspect is how individuals react and their willingness to make corrections. A possible component that became evident in the interviews is that no systematic contact was sought with colleagues or other specialists during pregnancy.

*‘No. I don’t speak to the gynaecologists. There is no exchange. If any, then with the doctors (PMR professionals) here at the centre.’ Jasmin Rieger, peer counselor*

*‘That was more one way, or it was at least not interdisciplinary. It wasn’t that a gynaecologist called me and said, “I have a patient here”, or that I spoke with the gynaecologist afterwards. The three patients whom I remember just now took things into their own hands—the communication. They got advice from me and then said, “Good, now I know, that’s enough.” The gynaecologist never called me about these patients, and I didn’t call the gynaecologist.’ Michael Brunner, urologist*

Cooperation and team effort were different during the phase of a forthcoming birth. In this case, we observed signs of cooperation, initiated especially by the attending specialist. The person in charge, usually the gynaecologist, called for team meetings with the anaesthesiologist, midwife, and nurse and informed his/her team of the impending birth.

*‘An epidural anaesthesia does not present a problem for the anaesthetist, and the woman’s handicap is also not a problem for the midwife. Of course we discussed it.’ Matthias Gerber, gynaecologist*

*‘Completely normal; there was a midwife during the birth, and as I said, caesarean section. We had arranged a discussion with the anaesthetist in advance. When there is a special existing disease, we don’t do that only a day before the planned caesarean section, but we plan things a little better in advance.’ Martin Roggo, gynaecologist*

#### Differences in the willingness to integrate competence of women with SCI in the care process

We observed that all participants had similar perceptions on how to handle medical health problems by participating in decision making. The interviewees recognized a high level of health literacy in the women with SCI. They described them as active and well-educated in health matters and as someone who searched for relevant information online or obtained information from their social surroundings. The interviewees also said that the pregnant women with SCI had a thorough knowledge of paraplegia, but insufficient knowledge about gynaecological and postnatal topics. The service providers with little knowledge about SCI often relied on the knowledge and arguments of the women with SCI and included them in the treatment processes and decisions.

*‘What occurred to me (is that) women who have given birth and sit in a wheelchair, they are like a mobile library. They know so much, one can really suck up their knowledge. I consider them to be the best sources of information.’ Anna Weiss, midwife*

#### Standards and scientific evidence

We consider that the guidelines produced by scientific specialist societies are of decisive importance for the treatments delivered by the service provider. The practice derives relevance from the description of medical standards. This knowledge is indispensable to enable service providers to avoid substantial treatment errors. They are practice-oriented aids for decisions concerning the course of treatment in the presence of special health problems. All study participants complained in general about the lack of guidelines, little existing scientific literature, and the lack of a central database.

*‘No, there is no knowledge and very little literature available. So, if you care for a woman with SCI, then you need to inform yourself.’ Anna Weiss, midwife*

*‘…urologists who are allowed to participate in the care of such patients do that with a good conscience—or at least I hope so. But nobody makes it transparent. There are, however, certain cooperation’s—the beginning of an exchange about different things. But there is no compilation of knowledge to provide a better basis for decision making.’ Michael Brunner, urologist*

It is usually accepted that the treatment provided by professionals is a mixture of their expertise, intuition, and the art of healing. The modern concept of evidence-based medicine demands that decisions be made whenever possible on the basis of empirically proven effectiveness. We experienced that the lack of easy-to-use tools forces professionals to rely on their specialist knowledge, which only partially covers the demands of treating para-gynaecological conditions. This inadequate knowledge certainly contributes directly to professionals’ uncertainty and can influence their willingness to provide medical care.

*‘Which therapies exist? What is allowed, and what is not? Where do we have experience, and where do we have none? I believe we simply have little experience. There are few statistically confirmed data on what is good and what is not good. We have to honestly admit that.’ Alexander Brand, urologist*

#### Healthcare organizations

It is known that access to appropriate medical care helps women maintain or improve their health. The study participants reported on the structural and architectural barriers fulfilling the needs of women with SCI.

Although most hospitals in Switzerland are equipped for persons with disabilities, we identified signs that the furnishings and equipment inside are not optimal for medical examinations of women with SCI. The interviewees initially had the impression that the practice or hospital would be entirely accessible and would meet the needs of disabled women. Only after specific requests with concrete examples did health professionals reflect on possible barriers. For example, there are no examination tables with adjustable heights, no scales, and the examination rooms are excessively narrow. In addition, the toilets are located on a different floor.

*‘Yes, that’s right, we don’t have scales.’ Martin Roggo, gynaecologist*

*‘Speaking of toilets – we do have a special toilet for people with disabilities. It isn’t on the same floor, but it is on a floor below.’ Matthias Gerber, gynaecologist*

Over and above the lack of documented knowledge, we found indications that the architecture of healthcare settings do not cater to para-gynaecological services in Switzerland. For example, rehabilitation centres can care for women with SCI during their pregnancy. However, they are not equipped for gynaecological treatments such as regular examinations during pregnancy.

*‘There isn’t anyone with exact knowledge about these things. Everyone knows a little bit. It is a very, very specialised topic. But there is no competent person who has knowledge of every aspect.’*

*‘We don’t have gynaecological equipment here, that’s right. But even if we had it, a specialist from gynaecology would have to do it.’ Nicole Müller, PMR professional*

*‘There is good access, one can use the examination rooms well, but then a gynaecologist is required who ideally would, for example, conduct preventive examinations. We don’t have that here, and not everyone can bring her own gynaecologist here. When a pregnant woman comes here and I want to do a urologic examination, it is absolutely no problem. But if I am not sure if there is also a gynaecologic problem, I cannot find that out here. Then I send her back to her treating gynaecologist, but I do not know and cannot influence how access is there, whether barriers exist to reaching the service. Sure, it’s not a problem here, but there is no gynaecologist here.’ Michael Brunner, urologist*

In summary, access to medical care cannot be regarded as patient-specific in this group of patients. The inadequate medical services offered can influence the choice of physician and add to the uncertainty faced during the provision of care.

### Strategy

#### Strategies applied to cope with uncertainty

We observed different mechanisms for coping with uncertainty. The different approaches, e.g., ‘…*in the worst case, I simply try to build a good working relationship with the gynaecologists*’ *Georg Fischer, PMR professional*, and *‘…close medical supervision…’ Martin Roggo, gynaecologist*, clearly indicate the different ways in which the participants deal with uncertainty. These strategies can be categorized into the following types: (i) *protective concerned attitude*, exhibiting great confidence in one’s own abilities usually based on long-term experience in the chosen specialty; (ii) ‘*no big deal’ attitude*, with a selective provision of information and the tendency to only react when problems arise; and (iii) *precautionary attitude*, which is characterized by many examinations.

#### Protective concerned attitude

A protective concerned approach to cope with uncertainty in the provision of care corresponds with medical care to protect the woman. The service provider radiates great reliability. This occurs without losing sight of optimal specialist care and a certain degree of quality of care by all involved parties. The characteristics of service providers from this category are focussed less on maintaining formal, logical treatment standards, but more on the ability to recognize patterns. The practitioners apply flexible treatment approaches based on clinical experiences.

‘*In principle, I would reduce antibiotic treatment as much as possible. Since the problem is common, and we see that even now, regardless of pregnancy, many patients with bacteriuria are treated, despite it being unnecessary. It is simply done; there are bacteria in the urine, and they are treated with an antibiotic. In a pregnant woman, especially if she is asymptomatic and shows no signs of a really complicated ascending infection, I would not give a long-term prophylaxis during the pregnancy.’* Alexander Brandt, urologist

We observed this kind of coping with uncertainty by service providers had long-term clinical experiences combined with leading positions.

*‘You know, in the worst case I simply try to build up a good working relationship with the gynaecologists. If the woman feels insecure, we have to discuss it. If possible, all three of us; otherwise only with the gynaecologists. We usually come up with a good solution, and things turn out well.’*

*‘I am always reassured when I have spoken to him (the gynaecologist) and when I see that he understands the problems or the possible problems.’ Georg Fischer, PMR professional*

With a protective concerned attitude, the aspect of high specialist proficiency comes to the fore accompanied by a curiosity to learn more about the problem. Most health professionals with this attitude endeavoured to find possible solutions for therapeutic interventions.

*‘What I have learned and my experience, in principle, I owe it to the patients. From the very beginning, my patients have told me things; I tried to remember and apply this to the next patients. All the tips and tricks I have learned have all been from the patients.’ Georg Fischer, PMR professional*

‘*In all the package inserts of drugs to treat bladder spasticity, it says that they are contraindicated in pregnancy. If you inform yourself more thoroughly and read up, there are apparently certain periods during pregnancy when this medication caused something in animal experiments. If you compare the doses that were given to the animals to what is administered to humans, the animals got higher doses, i.e. it says apodictically that one must not give it, but if you look at what that’s based on and take the trouble to do that for every single medicine, you will find out that that it is not apodictically true. But these data are very, very difficult to obtain; there is hardly any transparency, e.g. from the manufacturers. I really took the effort until I got any data at all from the different manufacturers. And then you have to see to what degree these data from animal experiments can be transferred to humans. There is little cooperation because there is also little interest on the part of the pharmaceutical companies that produce the medication.’ Michael Brunner, urologist*

#### ‘No big deal’ attitude

The ‘no big deal’ attitude is practiced by service providers who provide services only for pregnant women. Pregnant women with SCI are not considered as special cases by these service providers, and they treat them like every other pregnant woman. Even if each pregnant woman with SCI is an individual case and this can be firmly anchored in the service provider’s memory, it generally becomes blurred in the multitude of cases. As new patients are always waiting to be seen, the attending physician rarely spends more time than necessary on individual cases. He intervenes with help and support only if the woman explicitly expresses the need or if the medical situation calls for it. The help rendered is limited to the physician’s specialty and involves hardly any support for SCI comorbidities in the sense of directly taking over responsibility for all health-related problems. Another aspect of the ‘no big deal’ attitude is the selective information behaviour of the service provider.

*‘…I consider the pregnancy as such to be unproblematic. She is just a patient like any other.’* Jonathan Steiner, gynaecologist

Interviewer: *‘Did she have several, did she have more frequent ultrasounds?’*

Interviewee: *‘No, absolutely not.’*

Interviewer: *‘You may have been confronted with diverse secondary complications, like urologic problems and problems with bowel management. How did you solve these problems? Did she simply inform you of them, or did she consult someone else, or how did you handle the situation?’*

*Interviewee: ‘It was never actually brought up.’ Jonathan Steiner, gynaecologist*

*‘I mention it when I notice that it presents a problem for the woman. I do not do it on principle, in one fell swoop, like with you now, that you hear everything at once.’ Elisabeth Frey, gynaecologist*

*‘One need not necessarily communicate the problems to the patient, perhaps not to frighten her, but you have to have it in the back of your head and can then gradually integrate it into the process if need be.’ Jonathan Steiner, gynaecologist*

#### Precautionary attitude

Service providers take over responsibility as a form of precautionary action, especially when expectations from them are perceived as exceptionally high. Our investigation revealed that those service providers with few such cases are overburdened with the specialist-oriented support. They attempt to cover the medical aspects of both the gynaecological and paraplegic fields. As a result, the service provider often has to master highly complex issues and must decide accordingly under conditions of high uncertainty. Self-proclaimed expertise and the resulting responsibility obligate one to practice conscientiously. The aspects of a precautionary attitude were more examinations and a systematic administration of preventive medication. This kind of behaviour can be understood as an avoidance manoeuver and a sign of helplessness in dealing with the treatment situation. The service provider tends to make an increased number of diagnoses to be sure to cover all eventualities. We observed this attitude in gynaecologists as well as PMR professionals.

*‘You are already relatively restrictive in normal pregnancies, or you will treat every pregnant woman with even the slightest sign of a urinary tract infection. This is truer for women with SCI. Then you are actually even stricter.’ Nicole Müller, PMR* professional

*‘We naturally see these patients at shorter intervals, by shorter I mean we perform a control check-up every 3–4 weeks to have the parameters better under control.’ Martin Schüssler, gynaecologist*

### Consequences

We have argued above that management in this particular situation can be understood as attempts to restore security. As a consequence, the professionals try to withstand the uncertainty or hand it over. Sometimes role allocation and responsibility were unclear during the treatment process, and the service provider saw him/herself as only a part of this process. This can be recognized in the call for interdisciplinary collaboration.

#### Withstanding or handing over uncertainty

The study participants tried to confront their lack of knowledge by withstanding the situation, acquiring further knowledge, or handing over responsibility and tried to re-establish certainty. Some attempted to reduce the feeling of uncertainty by referring the pregnant woman with SCI to a different institution or by taking a lot of precautions. Others withstood the uncertainty and tried to better protect themselves by maintaining collaborations or networking with other professionals.

*‘What else can you do? So that I don’t just stand there like a fool. You thought over what you would do for a bladder infection. Can you do something preventive? Can you do something against obstipation? Can you do something to reduce the risk of thrombosis? And so on.’ Matthais Gerber, gynaecologist*

*‘For patients who have questions concerning bladder-sedating medication, there is no reliable answer because all these medicaments carry a theoretical risk, which must be prevented during pregnancy. I always gave a conscientious answer according to my best knowledge after consulting with other urologists. That satisfied the patients, and nothing happened to the children. That’s the best we can say.’ Alexander Brandt, urologist*

The difficulties and challenges that the professionals have to overcome in addition to all other expectations of medical service can be considered as burdens. An initial open and positive attitude toward the treatment of a pregnant woman with SCI could change during treatment. A midwife, after reflecting, expressed the wish to hand over responsibility for pregnant women with SCI.

*‘…just someone who can share his experience or even just cares for the women from beginning until the end. Without upcoming doubts or uncertainty. Or, as I said, somebody who has the knowledge and can pass it on or spread the knowledge. That would be great!’ Anna White, midwife*

#### Actor in a fragmented care process

A further consequence was the individual interaction with other professionals providing specialist treatment. Professionals especially from the field of spinal cord medicine were left in the dark with respect to how their treatment had affected the pregnancy and the child. Sometimes, they were also confronted with the women’s frustration resulting from being expected to make the decision concerning a medical problem by themselves. Professionals responsible for specialist treatment found themselves in an area of conflict among fragmented treatments, pregnant women requiring information, and their uncertain role in the treatment process.

*‘The gynaecologist will tell her something about pregnancy. We will tell her something urologic and maybe something about possible complications. But we do not sit down together, and then she’s there and asks herself, ‘What should I do now?’ The gynaecologist said everything is easy going just like anybody else, although maybe he has only seen one or two such women in his life. In principle we say that, as far as we know, the women we have seen are fine, but there are these and these risks.’ Alexander Brand, urologist*

#### Call for interdisciplinary collaboration

The increasing number of specialisations in our healthcare system has made a holistic view of the patients more demanding. What is needed is, therefore, an interdisciplinary collaboration by health professionals of all specialisations. The topic of interdisciplinary collaboration with respect to the supervision of pregnant women with SCI was broached in this context. We interpret the desire, above all among PMR professionals, for more interdisciplinary collaboration, even education from competency centres, as a result of the lack of institutionalized cooperation up until now. Interdisciplinary collaboration would liberate service providers from having to make decisions alone.

*‘Pregnancy and paraplegia, that’s not an everyday situation. Not every gynaecologist can deal with it, maybe not even every urologist. It is not very common even in specialised centres for SCI. I believe, perhaps an idea would be if one simply said, ‘OK, let’s combine them in a certain centre’….because I certainly lack some gynaecological aspects, I have to admit it. I know I cannot administrate that drug, I should not prescribe it. We should simply collaborate together, also for infections. The gynaecologist may use a dipstick and say, ‘Good, it’s positive; we have to treat her. I must give her a prophylaxis.’ We see that differently from a urologic point of view. I believe it would make sense to say, ‘OK, we put them all together in a centre and generate experience in a centre for that kind of thing.” Alexander Brand, urologist*

*‘I also believe that gynaecologists should know that they are dealing with special cases and that they also (should) enter into collaboration with rehabilitation centres. I do not think that someone in an outpatient clinic, a general practitioner, can do that. I do not think so. But, as I said, we are not gynaecologists, i.e., patients also need a gynaecologist. There simply must be cooperation, also after pregnancy.’ Julia Peter, PMR professional*

## Discussion

This study presents insights into the experiences of health professionals in caring for women with SCI during pregnancy and childbirth. The professionals reflected on a number of issues that affected their care delivery and interactions with pregnant women with SCI as well as with colleagues and allied health professionals.

The emergent core category was *delivering care under uncertainty in a rare event* (Fig. 1). The *rarity of the event* is characterized by the absence of specialised providers, few scientific publications, and a lack of specific sources of information and appropriate standards. Another emergent term in our core category was *uncertainty*. Uncertainty has been researched in various disciplines, including sociology [38, 39], psychology [40, 41], health services research [42], and patient counselling [43], and it provides several conceptual meanings that are in many cases not that different [44–46]. We share the view of Babrow [47], who states that uncertainty exists when details of situations are ambiguous, complex, unpredictable, or very probabilistic; when information is unavailable or inconsistent; and when people feel insecure in their own state of knowledge or the state of knowledge in general. Uncertainty in healthcare is experienced by not only clinicians but also patients [44, 48].

In people with SCI, subjective symptoms can be attributed to numerous health problems [49]. The trajectory of the pregnancy varies substantially among women [14], and as the study results indicate, some treatments can be considered experimental. Healthcare providers and patients both encounter complexity and ambiguity in decisions about diagnoses and treatments, which later on may be proved false. The aetiology of health states comprises various possible biopsychosocial factors; symptoms may be attributed to different paraplegic comorbidities or unknown causes, and healthcare providers may offer different diagnoses when assessing the same symptom pattern. The complexity endangers the healthcare professionals’ ability to treat and function optimally, which is closely associated with the ability to control and predict care situations.

The three strategies identified in this study, i) protective concerned attitude; ii) no big deal attitude; and iii) precautionary attitude, are seen as different ways in which health professionals manage the complex care situation. Identifying strategies [50] offers a possible explanation for how health professionals handle uncertainty and complexity. It provides a contextual framework from which to view clinical decision making. Although health professionals may not be able to come up with a clear-cut diagnosis, they could deal with patients’ concerns by talking about the probable diagnoses or by alleviating fears about treatments and restoring a certain level of security. The identified strategies can also reveal approaches and methods for problem solving.

The contextual conditions identified in this study (Fig. 1) are not only directly related to the core phenomenon. They may have also implicitly influenced the set of attitudinal responses of health professionals to the resulting phenomenon. In our view, the uncertainty of health professionals stems from different reasons and is related to the structural and architectural difficulties in accessing medical care, the lack of standards, and poor scientific literature as well as the behaviours of individual health professionals. Similar contextual findings have been observed and discussed as barriers in earlier research, e.g., inaccessible healthcare provider offices in general for mobility-impaired people and unfamiliarity of specific providers with fertility, pregnancy, and SCI [51, 52]. Equitable healthcare cannot be guaranteed under these conditions [53].

Some recommendations for the treatment or monitoring of women with SCI during pregnancy and childbirth are available in English-speaking regions [19, 54–56]. The German medical association for spinal cord injury (Deutsche Medizinische Gesellschaft für Paraplegie) is currently working on recommendations in German. Unfortunately, thus far, none of the available information seems to have reached healthcare professionals.

This has serious implications on care delivery. Possible paraplegic comorbidities that might arise during a pregnancy with SCI remain unknown to professionals. Preventive measures are not applied routinely on the patient. Additionally, recommended preventive measures and diagnostic and laboratory tests may not be carried out. A previous study demonstrates that 10 of 17 women were hospitalized during the course of their pregnancies [20]. The high number of hospitalizations indicates possible unfavourable decisions due to insufficient knowledge. These findings underline the importance of clinical research and the need for favourable ‘daily’ clinical training of health professionals on as many cases as possible in order to ensure optimal health for the mother and child.

The lack of specific experts and care programs for women with SCI during pregnancy and childbirth pose a serious question for health professionals: to what extent should they want to take over responsibility. Care delivery took place in two diverse medical specialties with different treatment philosophies, and the interviewees differed in their views on who should take the overall lead. On one side, we had gynaecologists and obstetricians specialised on an organ system or event. On the other side are professionals from the field of rehabilitation who are trained in delivering care for multiple organ systems and even psychosocial care. The different professional understandings affected the role and responsibility of how health professionals see their position in the care process for pregnant women with SCI.

The results of this study indicate a need to integrate multiple professional perspectives in the care process in order to optimize care and treatment to people where fragmentations in care had led to unfavourable care experiences and outcomes [14].

It became apparent that we face a structural problem. Health professionals support the idea of *‘centralized’* care, whereas women with SCI favoured receiving care in their local environment as identified in a previous study. Pregnant women with SCI did not prefer travelling to a specific centre. Moreover, they did not like the idea of receiving care in a rehabilitation setting for their pregnancy concerns [14]. On the other hand, the study participants mainly from the rehabilitation settings felt like a puzzle piece in the care process. One solution is to create a best practices model of how healthcare for women with SCI during pregnancy and childbirth could be performed at a top level and to enter in a stakeholder dialogue [57] with all groups. A recent study demonstrated that the effectiveness of integrated care projects is improved when all stakeholders perceive a high degree of integration. This emphasizes the need to develop a multilayer commitment when leading integrated care efforts [58]. In the meantime, virtual places such as SCI parenting.com from the Spinalis Group in Sweden [59] or the guide for pregnancy with SCI of the Spinal Cord Injury Perinatal Interest Group (Rick Hansen Institute) from Canada [60] could serve as a case in point for the German-speaking region.

Another option is creating a clinical quality management system [61] that provides a framework for defining and delivering quality outcomes, managing risks, and continual improvement. The system should be set up as a learning system to define parameters that could be important for creating a database for pregnancy outcomes.

### Study limitations

The qualitative methodology allowed us to gather in-depth views on how health professionals experienced care delivery for women with SCI during pregnancy and childbirth. We decided to conduct individual interviews with the experts instead of focus groups or written interviews assuming that besides time constraints, the theme may not be that important to health professionals due to the limited overlap with their specialty. We aimed to increase the chances of participation by providing participants with the choice of an oral or telephone interview and allowing them to choose the location of the interview.

During expert recruitment, we had to consider the busy time schedule of the participating experts. Therefore, the interviews were limited in scope of potential for openness, possibly affecting the quality of information [62]. Face-to-face interviews would have been preferable in all cases, allowing for a better rapport and increased sensitivity to the more subtle issues at stake. Since interviews were conducted by well-trained researchers, we are confident of having gathered data on sensitive issues as well. The first author is a paraplegic women who is familiar with SCI problems; enabling her to be sensitive both to her research perspective and to the situation of women with SCI. The research assistant learned continuously by assisting the initial interviews and shaped own interviews with her special interest for healthcare services use. Each interview style has advantages and disadvantages, and the differences eventually enriched the picture of health professionals’ experiences. The assistants’ lack of experience with SCI conditions sometimes encouraged the interviewees to explain in further detail. Additionally, the first author challenged health professionals by using concrete examples from women with SCI and encouraged them to speak up openly about their own experiences.

In qualitative research, the question of the extent to which analyses are affected by the researchers’ own values and objectives and its effect on the research project always arises [63]. We aimed to reduce the influence of single researchers’ views by including different perspectives. The analysis was based on continuous discussions between the authors and research assistant throughout the process in order to increase the scrutiny and trustworthiness of the analysis. Moreover, the credibility of the results was enhanced by thoroughly reading and analysing the data during open and axial coding separately by two researchers before comparing and confirming the categories.

Since the sampling strategy was based on a previous study in which women named their treating health professionals, certain groups of importance in other healthcare systems, such as family doctors and neurologists, may not appear. This deliberate choice of focussing only on the known groups treating pregnant women with SCI may mean that certain perspectives were lost. This might have led to a restriction of possible experience from the professional side, albeit representing the current care situation of the women with SCI involved.

The small sample size does not allow for substantive comparisons between professional groups. Therefore, we primarily restricted our interpretation the group of professionals as a whole. During the analysis process, we had indications that health professionals’ behaviour was primarily influenced by their experience and perceived role and not especially by their specialty. Therefore, we highlighted these aspects in the results.

Although the results refer primarily to the healthcare situation of pregnant women with SCI in Switzerland, the conceptual design of the core category allows a transfer to other special situations, such as multiple sclerosis and spina bifida, which are distinguished by a rare event and medical uncertainty.

## Conclusions

These findings highlight the diversity of perspectives among different health professionals regarding the approach to care and service delivery for pregnant women with SCI. There is a need for specific services, more information, guidance, and guidelines for health professionals caring for woman with SCI during pregnancy and childbirth. By drawing upon the study results and discussing possible solutions, we tried to provide realistic and feasible solutions, bearing in mind that the presented solutions may also be valid for a wider field that includes different disabilities as mentioned above. A centre of excellence not primarily for care, but for reference, which collects and evaluates clinical experiences with pregnant women with SCI may be a valuable aid for health professionals involved in the medical care of these patients. Alternatively, contradicting the wish of women, an interdisciplinary centre offering specialised care could be worthwhile solution.

While it is important to better comprehend the complex and multidimensional nature of the rare event, it is also important to create integrated care designs in terms of promoting coordination and continuity of care and equity of access. We strongly recommend further prospective research on the development of integrated care concepts and support research with clinical impact, i.e., clinical studies on the subject to provide a broader evidence base for decision making and, ultimately, for reducing uncertainty for all stakeholders in this rare event.

## Abbreviations

PMR, physical and rehabilitation medicine; SCI, spinal cord injury; GT, grounded theory.

## Additional file

Additional file 1: Interview guide. (DOCX 28 kb)
