# Supplementary material for: Delivering care under uncertainty: Swiss providers’ experiences in caring for women with spinal cord injury during pregnancy and childbirth – an expert interview study
Source: BMC Pregnancy Childbirth. 2016 Jul 22;16:181. doi: 10.1186/s12884-016-0976-y (PMC4957428; doi:10.1186/s12884-016-0976-y)
Supplement: Additional file 1: — Interview guide. (DOCX 28 kb) [file 12884_2016_976_MOESM1_ESM.docx]

## Semi-structured Interview guide

*Personal or telephone interview*

*(translated from German to English)*

***Phone interview***

*Hello,*

*We are conducting a study about pregnancy and childbirth in women with a spinal cord injury. Do you have delivered care to a woman with a spinal cord injury during pregnancy or birth? May I ask you some questions about it? Either I visit you or if it would be more convenient for you, we could also arrange a phone interview. The interview will last about 30 min. When can we see us or when can I call you again?....*

*The next days you will receive by mail additional information about the study and we will also reconfirm the interview date.*

***Interview***

*Thank you for participating in our study. I briefly summarize the study goals.*

*The aim of the present study is to examine the health care services situation in women with SCI during pregnancy and childbirth in Switzerland. In this context we will conduct different studies and this one is with health care professionals.*

*We ask health professionals about the medical needs of women with SCI during pregnancy and childbirth; which factors influenced a health service use; which services were used and how you experienced the entire care with all involved stakeholders.*

*In this context I will now ask you some questions.*

Warm up

- Could you shortly introduce yourself? Your name, specialty field, where you work and maybe how many women with SCI you have seen.

Medical challenges - Need

- Was the woman we talk about your only patient with SCI or did you treat other women with SCI or another disability?
- Which medical needs did the women have? Why did she visit you?
- What do you think, what are the general needs of women with SCI or women in wheelchair during pregnancy and childbirth?

Health Services Use (Type, Setting)

- Which services did you offer to the pregnant woman with SCI?
- How did you experience the care of women with SCI? Was it different to women without SCI? If yes, what was different? If no: How would you explain that?
  - (reasons: immobility, assistive devices, care effort, associated medical problems, etc.)

I suppose treatments took place in your practice/hospital.

Contextual factors

- How would you describe the accessibility to your practice/hospital for women in wheelchair?
  - (Entry, parking places, toilets, examination room, examination tables, changing room, etc.)
- Were there any other factors, which could have influenced the care ?

(For example, was the women’s knowledge (health literacy) about possible complications of any advantage ? Did the insurance coverage had any influence? Or, was everything always clear and easy? Guidelines and information possibilities?

- Was the women already your patient before her pregnancy?

Expertise - collaboration

- How did you experience the collaboration with other health professionals?
- Did you seek help of one of your colleagues? Or did you request additional expert opinion? If so, where? Was the information satisfactory? How did you inform yourself about possible risks ?
- Did you refer a patient to a colleague? If yes, why?
- Did you collaborate with a hospital/expert? If yes, did you experience the collaboration with them?
- Could you name an institution or person to which health professionals (physicians, midwives or nurses) could seek advice or address their questions about paraplegic women during pregnancy?

Suggestion

- How could health services for pregnant women with SCI be improved ?

Final questions

- Are there any other experiences or impressions that you would like to share with us about providing care for a paraplegic pregnant women ? Or did I forgot to ask you something?
- Would you again provide care for a women with SCI during pregnancy and childbirth?

*Thank you for your time and sharing your experiences with us!...*
